# Intelligent metasurfaces can recognize objects

**DOI:** 10.1038/s41377-022-00902-9

**Published:** 2022-07-07

**Authors:** Mengxin Ren, Jingjun Xu

**Affiliations:** The Key Laboratory of Weak-Light Nonlinear Photonics, Ministry of Education, School of Physics and TEDA Applied Physics Institute, Nankai University, Tianjin, 300071 China

**Keywords:** Micro-optics, Integrated optics

## Abstract

An on-chip optical neural network is built using metasurfaces, which can recognize objects with high accuracy.

## Abstract (web summary)

An on-chip optical diffractive neural network is built based on metasurfaces, which can recognize objects.

## Graphical abstract

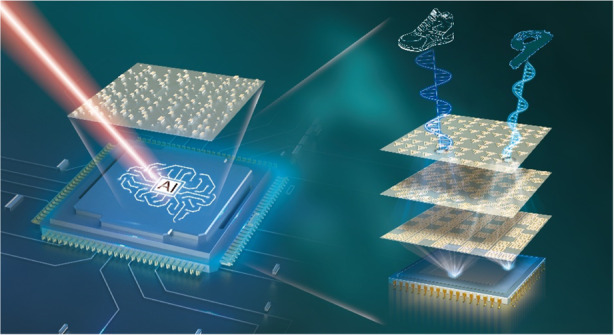

Photon is a perfect information carrier. Every day, countless photons travel to every corner of the world at a speed far exceeding that of any other substances. They run together or intersect without interfering with each other. Therefore, it is believed that the photonic systems can, in principle, handle thousands of information channels in parallel, which present a great computing capacity and communication bandwidth far beyond the electronic devices.

In fact, the human body itself is an excellent optical processing system. Billions of bits of visual information flood into our retinas in each second, and travel through the nervous system into our brain for processing. This super information processing ability has greatly enriched the fun of our lives, which nevertheless in turn stimulate our thirst for more information. It is increasingly recognized that only the parallelism of light can meet the explosive growth in our demand for information. As it turns out, almost all the information we get today comes from the photons in optical (or microwave) networks.

The initial idea of constructing an architecture, which uses photons instead of electrons for information processing, emerged shortly after the invention of laser. One straightforward implementation of such idea is pattern recognition, which is essentially the basic function of the biological eyes. For this purpose, optical cross-correlators based on Fourier convolution were proposed and built^1^. During the past decades, the concept of artificial neural networks has attracted much attention because it shows high recognition accuracy even for complex objects^2^. Recently, optical neural networks have become a hot pursuit due to their high speed, high parallelism and low energy consumption compared with the electronic counterparts. As a demonstration, the all-optical diffractive neural networks have been demonstrated at terahertz wavelengths^3^. However, it is highly desirable to realize similar devices in visible range, which is a direct mimic of our visual system.

Now, writing in this issue of *Light: Science & Applications*^4^, Luo et al. realize an on-chip optical diffractive neural network using metasurfaces in the visible spectral range, which can recognize objects with a high accuracy (Fig. 1). The metasurfaces are two-dimensional thin-film planar structures, which forms a new framework for ultracompact photonics devices. By engineering their building blocks named as meta-atoms, the metasurfaces demonstrate an unprecedented flexibility in controlling light field^5^. Due to the subwavelength feature of the metasurfaces, the scalar diffraction theory widely used in conventional discrete optical correlator is no longer applicable in principle. Full vector analysis must be adopted to reproduce the light behaviors within the nanoscale metasurfaces. Despite of mathematical sophistication, the vectorial nature of the light-matter interactions provides a possibility of optical multiplexing, which is helpful to increase the number of channels and enlarge the capacity of the neural network.

**Fig. 1:**
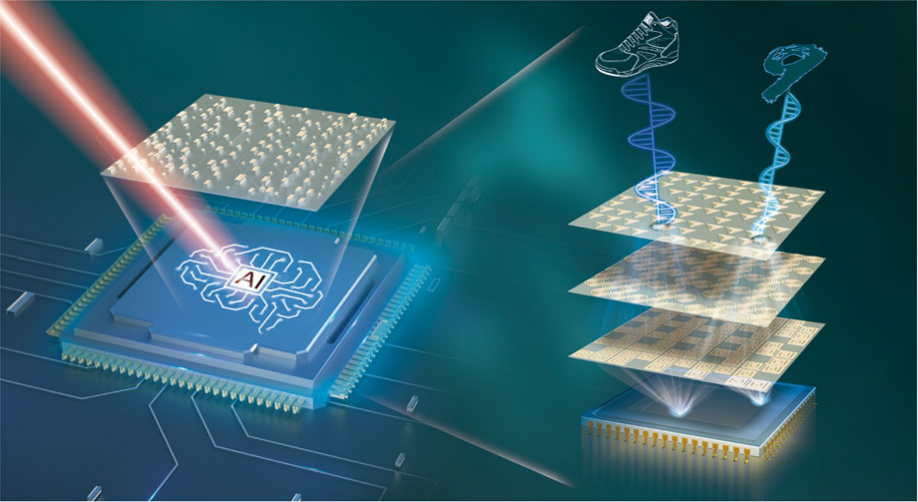
Schematic of metasurface-based on-chip optical neural network. The neural network is built using a polarization-multiplexed metasurface integrated with a CMOS imaging sensor. Different objects such as handwritten digits and fashion items can be recognized

In the present paper, the authors adopt a polarization multiplexing scheme, in which the optical information carried by orthogonal polarizations can be handled simultaneously and independently without interfering with each other. Such polarization multiplexing merit is achieved here by rectangular structural design of TiO_2_ nanopillar within one meta-atom, resulting in birefringent response for linearly polarized light. The nanopillars acts as neurons that interact with each other through optical scattering/diffraction. By varying the structural sizes of the nanopillars, both the local response and the inter-neuron coupling could be altered effectively. Thus, the response of the neural network is tuned effectively resulting in different output. To find the optimum network parameters, the authors trained the network using the cross-entropy loss as a loss function, which is often used in machine learning for object classification. The neurons in the network, are updated by a stochastic gradient descent algorithm. Finally, the geometric parameters of each meta-neuron are determined, and the metasurface layer is fabricated using a standard E-beam lithography technique.

The authors further integrate their metasurface onto a complementary metal oxide semiconductor (CMOS) imaging sensor, constructing an ultracompact artificial intelligence (AI) chip. In the work presented here, the authors manufactured the metasurface neural network that remembers two different kinds of objects: handwritten digits (0, 1, 3, 9) and fashion items (T-shirts, sneaker, trouser, and ankle boots). These two kinds objects are encoded into different polarization channels. After interacting with the metasurface, the incident light carrying the profile information of the target is diffracted onto a specific region of the CMOS sensor. By resorting to the correspondence between the region and the remembered objects, the target can be recognized. The AI chip here achieves a high recognition accuracy of greater than 93%. Benefitting from the subwavelength size of the metasurface unit cell, the areal density of the artificial neurons reaches 6.25 × 10^6^/mm^2^. Furthermore, because the fabrication of both the metasurface and the imaging sensor is CMOS-compatible, thus the AI chip here can be easily mass-produced by state-of-the-art semiconductor foundries.

It could be expectable that apart from the polarization multiplexing adopted here, many other multiplexing schemes can be used, such as wavelength multiplexing, spatial multiplexing, and vortex multiplexing, etc., which can substantially expand the processing channels of the neural network. Moreover, it worth noticing that the presented architecture is pretrained, in which the parameters of the meta-neuron have been fixed during the fabrication process. By introducing nonlinearities into the meta-atoms, the neuron can become reconfigurable and “alive”^6^. As a result, the properties of the neural network can adjust automatically, enabling self-learning. Furthermore, it would be interesting to extend the design in this paper to realizing optical convolutional^7^ or attention-based neural networks^8^, which can handle the recognition of highly complicated objects. To be a summary, the work presented here is certainly a firm step toward the “mind at light speed”^9^.

## References

[1] Goodman, J. W. *Introduction to Fourier Optics* 2nd edn (McGraw-Hill, 1996).

[2] Krogh A (2008). What are artificial neural networks?. Nat. Biotechnol..

[3] Lin X (2018). All-optical machine learning using diffractive deep neural networks. Science.

[4] Luo XH (2022). Metasurface-enabled on-chip multiplexed diffractive neural networks in the visible. Light.: Sci. Appl..

[5] Yu NF (2011). Light propagation with phase discontinuities: generalized laws of reflection and refraction. Science.

[6] Ren MX (2017). Reconfigurable metasurfaces that enable light polarization control by light. Light.: Sci. Appl..

[7] Hinton GE, Salakhutdinov RR (2006). Reducing the dimensionality of data with neural networks. Science.

[8] Vaswani, A. et al. Attention is all you need. *Proceedings of the 31st International Conference on Neural Information Processing Systems* 6000–6010 (Curran Associates Inc., 2017).

[9] Nolte, D. D. *Mind at Light Speed: A New Kind of Intelligence* (Simon and Schuster, 2001).

